# Kinetics, thermodynamics, equilibrium, surface modelling, and atomic absorption analysis of selective Cu(ii) removal from aqueous solutions and rivers water using silica-2-(pyridin-2-ylmethoxy)ethan-1-ol hybrid material†

**DOI:** 10.1039/d1ra06640d

**Published:** 2021-12-22

**Authors:** Said Tighadouini, Smaail Radi, Othmane Roby, Imad Hammoudan, Rafik Saddik, Yann Garcia, Zainab M. Almarhoon, Yahia N. Mabkhot

**Affiliations:** Laboratory of Organic Synthesis, Extraction and Valorization, Faculty of Sciences Ain Chock, Hassan II University, BP: 5366 Casablanca Morocco tighadouinis@gmail.com othmaneroby1@gmail.com hammoudanimad18@gmail.com rafik.saddik@gmail.com; University Mohammed First, Faculty of Sciences, Laboratory of Applied Chemistry and Environment (LCAE) 60000 Oujda Morocco s.radi@ump.ac.ma; Institute of Condensed Matter and Nanosciences, Molecular Chemistry, Materials and Catalysis (IMCN/MOST), Université Catholique de Louvain Place Louis Pasteur 1 1348 Louvain-la-Neuve Belgium yann.garcia@uclouvain.be; Department of Chemistry, College of Science, King Saud University P.O. Box 2455 Riyadh 11451 Saudi Arabia zalmarhoon@ksu.edu.sa; Department of Pharmaceutical Chemistry, College of Pharmacy, King Khalid University P.O. Box 960 Abha 61421 Saudi Arabia ygaber@kku.edu.sa

## Abstract

The removal of heavy metals is attracting considerable attention due to their undesirable effects on the environment. In this investigation, a new adsorbent based on silica functionalized with pyridin-2-ylmethanol (SiPy) was successfully synthesized to yield to a hybrid material. FTIR, SEM, TGA, and specific surface area analysis were used to characterize the structure and morphology of the SiPy hybrid material. Various heavy metal ions such as Cu(ii), Zn(ii), Cd(ii), and Pb(ii) were selected to examine the adsorption efficiency of the newly prepared adsorbent, optimized at varying solution pH, contact time, concentration, and temperature. The adsorbent SiPy displayed good adsorption capacity of 90.25, 75.38, 55.23, and 35.12 mg g^−1^ for Cu(ii), Zn(ii), Cd(ii), and Pb(ii), respectively, at 25 min and pH = 6. The adsorption behaviors of metal ions onto the SiPy adsorbent fitted well with the pseudo-second-order kinetic mode and the isotherm was better described by the Langmuir isotherm. The thermodynamic studies disclose spontaneous and endothermic adsorption process. Furthermore, the SiPy adsorbent retained good selectivity and regeneration properties after five adsorption–desorption cycles of Cu(ii). A computational investigation of the adsorption mechanism indicates that the *N*-pyridine, *O*-hydroxyl, and ether O-atoms play a predominant role during the capture of Cu(ii), Zn(ii), Cd(ii), and Pb(ii). This study proposes the SiPy adsorbent as an attractive material for the selective removal of Cu(ii) from real river water and real industrial wastewater.

## Abstract 2

The removal of heavy metals is attracting considerable attention due to their undesirable effects on the environment.

## Graphical abstract

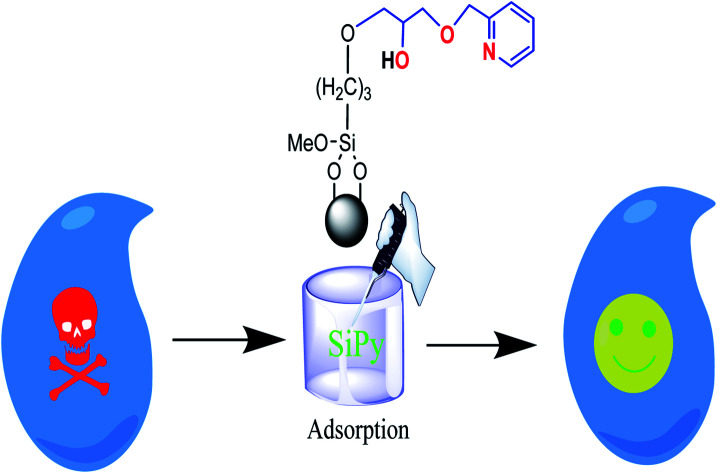

## 1. Introduction

With intensive industrial activity and urbanization, aquatic environment contamination by heavy metal ions has become one of the major environmental pollution sources of groundwater and surface waters^1,2^ since heavy metal ions are not biodegradable, and tend to accumulate and remain in living organisms for long periods, causing various disorders such as skin and liver diseases.^3,4^ Copper, zinc, cadmium, and lead are the most hazardous metals listed on the US Environmental Protection Agency's list of priority pollutants. However, their use cannot be avoided because of their necessity in tanneries, batteries, paints, coatings, dyeing, textile mill, and other growing industries.^5^ These industries release heavy metal ions into the environment and polluted water is used for irrigation and consumption.^6^ It has become essential to search for new methodologies to extract them from environmental aqueous solutions.

In recent years, various techniques and technologies were used for the removal of heavy metal ions such as ion exchange,^7^ reverse osmosis,^8^ chemical precipitation,^9^ membrane filtration,^10^ photocatalysis methods,^11,12^ filtration,^13^ electrochemical technology,^14^ solvent extraction,^15^ and flocculation.^16^ However, these techniques are limited in their practical use due to the generation of hazardous by-products.^17^ Adsorption is however considered a promising and effective purification technique for heavy metal ions due to its simplicity, low cost, and high efficiency.^18^

Conventionally, adsorbents such as carbon nanotubes,^19^ zeolites,^20^ chitosan,^21^ and cellulose^22^ have gained attention due to their promising potential in environmental domains. Mesoporous silica, however, offers a number of assets such as a large specific surface area, high thermal and mechanical stabilities, as well as regular porosity and flexibility toward surface chemical modification.^23,24^ In this respect, mesoporous silica gel can be easily functionalized to significantly increase the sorption of heavy metals ions from wastewater.^25–32^ The efficiency and selectivity of these adsorbents partly rely on the donor atoms such as oxygen, nitrogen, and sulfur onto the surface of the material that are responsible for forming a complex with heavy metals.^33–37^ In this context, pyridine has attracted attention because of its structure and well-known coordination chemistry.^38,39^ Pyridine is thus an excellent grafting group for functionalizing silica and can be widely applied in environmental cleanup *via* the chelating reaction.^40–42^

In this work, a silica gel impregnated with a ligand-based functionalized pyridine was synthesized and characterized for the extraction of heavy metal from aqueous solutions. On the other hand, density functional theory (DFT), noncovalent interaction (NCI), quantum theory of atoms in molecules (QTAIM) approaches, and the localized orbital locator (LOL) were employed to gain a better understanding of the metal ion's adsorption mechanism and selectivity of the ligand structure.^43^

## 2. Experimental section

### 2.1. Materials and methods

All solvents and chemicals (Sigma-Aldrich, purity 99.5%) were of analytical grade and used without further purification. Silica gel (Sigma-Aldrich) with particle size in the range of 70–230 mesh, surface area of 470–530 m^2^ g^−1^, pore diameter of 52–73 Å, and pore volume of 0.7–0.85 cm^3^ g^−1^ was activated before use by heating at 120 °C for 24 h. The silylating agent 3-glycidoxypropyltrimethoxysilane was used without purification. All metal ion quantities were determined by atomic absorption spectroscopy (AAS) using a Spectra Varian A.A. 400 spectrophotometer. The pH value was controlled using a pH 2006, J. P. Selecta; elemental analyses were performed by the Microanalysis Centre Service (CNRS). FT-IR spectra were obtained using a PerkinElmer System 2000. SEM images were obtained on a FEI-Quanta 200. Mass loss determinations were performed in a 90 : 10 O_2(g)_/N_2_(g) atmosphere on a PerkinElmer Diamond TG/DTA at a heating rate of 10 °C min^−1^. The specific surface area of modified silica was determined using the BET equation. The nitrogen adsorption–desorption was obtained by means of a Thermoquest Sorptomatic 1990 analyzer after the material had been purged in a stream of dry nitrogen.

### 2.2. Synthesis of pyridin-2-ylmethanol (L_1_)

To a mixture of LiAlH_4_ (5.66 g, 0.14 mol) and dry THF (90 mL), ethyl picolinate was added (6 g, 39.69 mmol) at 0 °C and the reaction was stirred at reflux for 4 h. After the reaction was completed, the mixture was cooled to 0 °C, and a solution of NaOH (15%; 5.66 mL) and distilled water (17 mL) were added successively. The resulting product was purified by column chromatography (CH_2_Cl_2_/MeOH, 9/1, silica) to afford compound L_1_ in 86% yield. Brown liquid; *R*_f_ = 50% (CH_2_Cl_2_/MeOH, 9/1; silica); IR (KBr, cm^−1^): *ν*(OH) = 3411; *ν* (C=N) = 1553; *ν* (C=C) = 1452; ^1^H NMR (DMSO-d6): *δ* 4.53 (s, 2H, –CH_2_); 5.38 (s, 1H, OH); 7.21 (m, 1H, Py-Hβ); 7.44 (d, 1H, Py-Hδ); 7.76 (t, 1H, Py-Hγ); 8.44 (d, 1H, Py-Hα); ^13^C NMR (DMSO-d6): *δ* 64.52 (1C, CH_2_); 120.60 (1C, Py-Cβ); 122.35 (1C, Py-Cδ); 137.02 (1C,Py-Cδ); 148.89 (1C, Py-Cα); 162.31 (1C, Py-Cε); MS: *m*/*z*, 110.05 (M + H)^+^.

### 2.3. Preparation of 3-glycidoxypropyl-functionalized silica (SiEp)

The surface modification of mesoporous silica material by 3-glycidoxypropyltrimethoxysilane (SiEp) was carried out according to our published method.^44^ Briefly, 10 g of activated silica was dissolved in toluene (40 mL), in which were added triethylamine (240 μL). Then, 3-glycidoxypropyltrimethoxysilane (13.3 mL) was added to the reaction and stirred at solvent reflux temperature for a period of 24 h under nitrogen atmosphere. The final product was filtered and extracted *via* Soxhlet extraction with ethanol and dichloromethane (1 : 1) for 12 h. The crude product was vacuum dried at 70 °C over 24 h.

### 2.4. Fabrication of pyridine-substituted silica (SiPy)

**Scheme 1:**
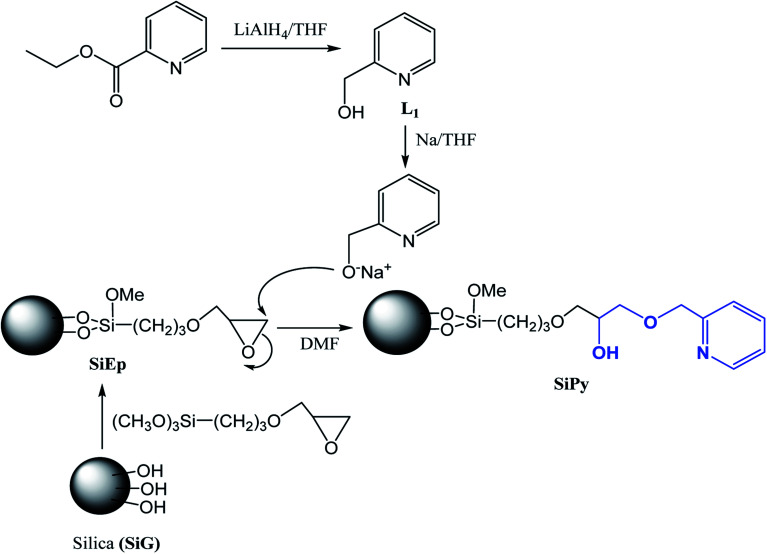
Synthetic route to prepare the hybrid material SiPy.

After converting the hydroxyl-pyridine (L_1_) (Scheme 1) to its alcoholate derivative (0.8 mmol) using Na/THF, it was added to SiEp (1.00 g) in DMF (30 mL). The mixture was stirred and refluxed for 24 h to give a brown solid (SiPy), which was purified using a similar procedure used for SiEp.

### 2.5. Batch adsorption experiments

The effect of concentration ([Mn(ii)] = 10 to 300 mg L^−1^), time (5 to 35 min), pH (1 to 7), temperature (25 to 45 °C), and selectivity (quaternary metal systems) parameters on the adsorption of Cu(ii), Zn(ii), Cd(ii), and Pb(ii) were studied in the batch method. Briefly, 10 mg of the SiPy adsorbent was dispersed to 10 mL of aqueous solutions. The pH values were adjusted with diluted HCl and NaOH solutions, the mixture was shaken at 25 °C for 25 min. After extraction, the metal ion concentrations were monitored by AAS and the adsorption capacity was calculated using eqn (1).^45^

1 *q*_e_ = (*C*_0_ − *C*_e_) × *V*/*W*

Here, *q*_e_ (mg g^−1^) denotes the adsorption capacity, *C*_0_ (mg L^−1^) and *C*_e_ (mg L^−1^) are the initial and equilibrium concentrations respectively, *V* (mL) refers to the volume of the solution, and *W* (mg) refers to the mass of the adsorbent.

### 2.6. Computational methods

DFT,^46^ QTAIM,^47^ and NCI^48^ utilizing GAUSSIAN09 (ref. 49) and Multiwfn^50^ softwares further validated the selectivity and mechanism of metal adsorption. The ligand was optimized using DFT based on Beck's three parameter exchange functional and Lee–Yang–Parr nonlocal correlation functional (B3LYP),^51^ combined with the 6-311G+(d,p) basis set,^52^ for M(ii) complexes to the basis LANL2DZ level.^53^

## 3. Results and discussion

### 3.1. Linker synthesis

The synthesis of the new material SiPy is presented in Scheme 1. The first step of the synthesis consisted of mixing activated silica gel with 3-glycidoxypropyltrimethoxysilane to form the material (SiEp). The second step consisted of the synthesis of the target ligand L_1_, which was converted to its pyridine alcoholate using metallic sodium in THF, which was then immobilized on the SiEp surface in DMF at reflux.^45^

### 3.2. Characterization of the material

#### 3.2.1. Elemental analysis

The result of the elemental analysis, determined for SiPy, showed high percentages for carbon (5.97%) and nitrogen (1.08%), which are not present on the starting silica. This result indicates that the organic matter (pyridine unit) has been immobilized on the silica network, thus supporting a successful functionalization. This immobilization of the organic matter on the inorganic silica network was also confirmed by the analyses below.

#### 3.2.2. FTIR characterization

**Fig. 1:**
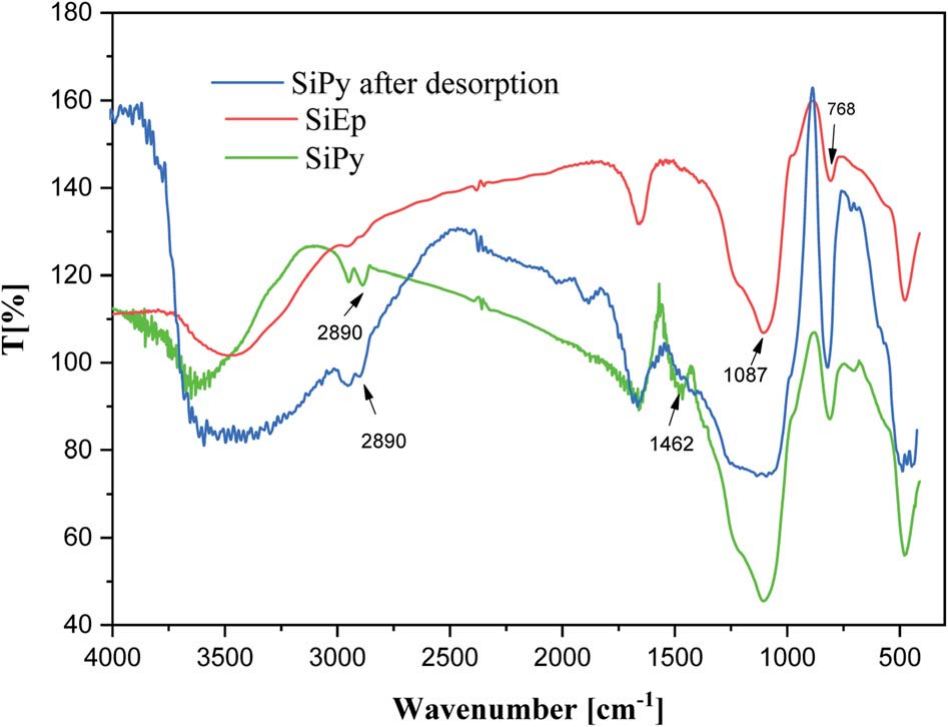
FTIR spectra of SiEp and SiPy (before and after desorption).

The specific peaks observed at 3440 cm^−1^, 1087 cm^−1^, and 798 cm^−1^ related to the silica backbone were identified.^54^ The SiPy material was analyzed using FTIR spectra and compared with pure silica SiG and SiEp(Fig. 1). After functionalization, the SiEp spectrum exhibits a new peak at 2890 cm^−1^, which originates from the C–H stretching bond. The spectrum of the final material (SiPy) showed a new peak at 1462 cm^−1^, which was attributed to the stretching vibration of C=N. This spectrum thus shows that the material was successfully modified by the pyridine derivative.

#### 3.2.3. Scanning electron micrographs (SEM)

**Fig. 2:**
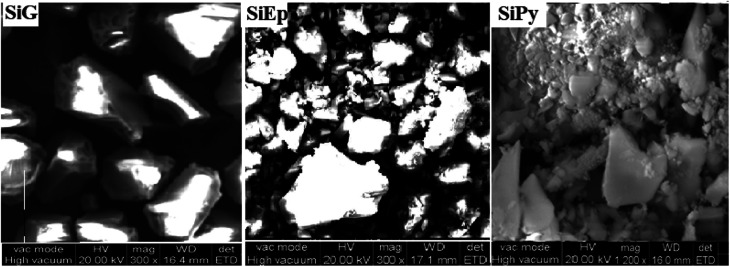
SEM micrographs of SiG, SiEp, and SiPy.

As we can see from SEM imaging (Fig. 2), the surface morphology of the non-functionalized and functionalized silica particles are clearly different. Indeed, an irregular and smooth surface was observed for silica particles (SiG), whereas a rough and porous morphology was observed for the hybrid material SiPy, where the agglomeration of molecules was much elevated, thus successfully confirming the modification of the silica surface.

#### 3.2.4. Surface properties

**Fig. 3:**
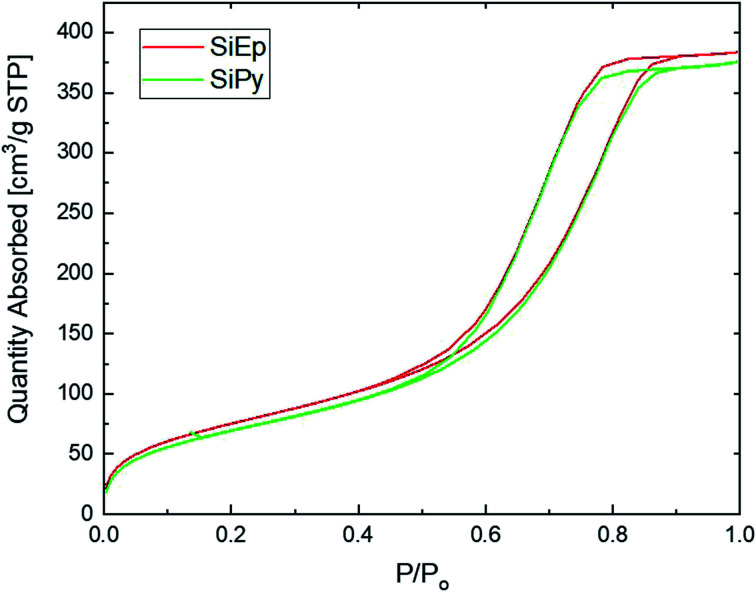
Isotherm of SiEp and SiPy.

While a porous structure was observed for SiPy, it was of interest to determine its eventual mesoporous character by nitrogen adsorption/desorption (Fig. 3). Such a character was confirmed by a type IV isotherm, and a visible hysteresis loop, indicating a H2-type profile based on theIUPAC classification.^55^ The surface area for free silica SiG is 305.21 m^2^ g^−1^ with a pore volume of 0.770 cm^3^ g^−1^. The BET surface area of SiEpand SiPy decreased to 277.08 and 261.33 m^2^ g^−1^, respectively, due to pore blocking by the chemical bonding of free silica with organic functional groups. It means that part of the pores were blocked by organic groups, which led to a decrease in the size of the pores. The total pore volume of free silica was 0.770 cm^3^ g^−1^ and it decreased to 0.68 and 0.66 cm^3^ g^−1^ for SiEp and SiPy, respectively.

#### 3.2.5. Thermogravimetric analysis (TGA)

**Fig. 4:**
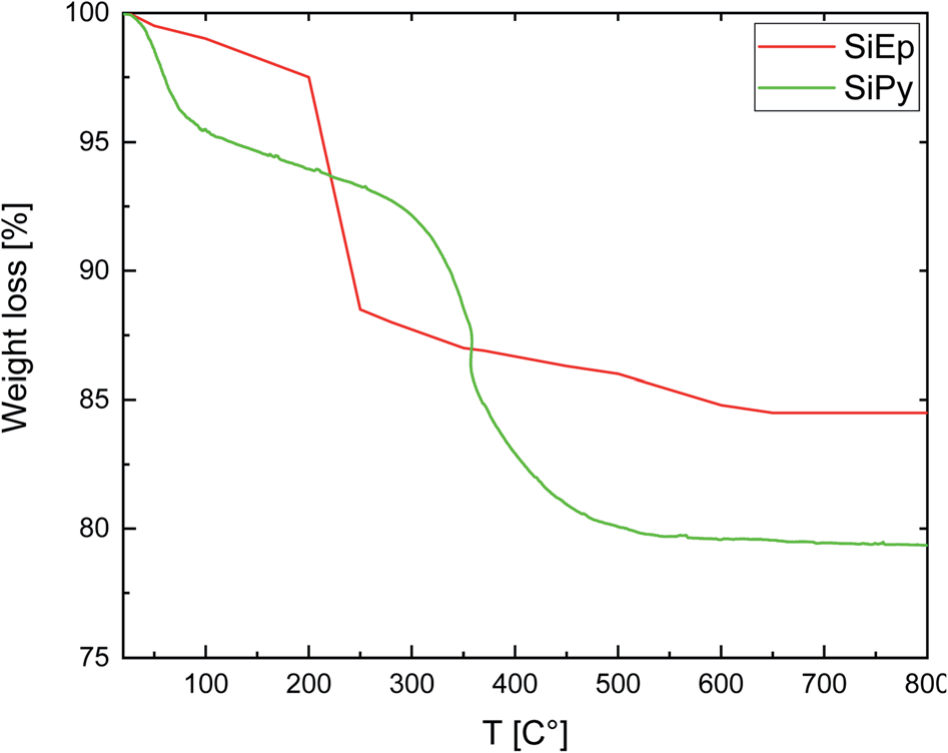
TGA data of SiEp and SiPy.

The thermal stability of SiG, SiEp, and SiPy was examined by TGA analysis (Fig. 4). SiG showed a weight loss of less than 3.15% from 25 to 110 °C due to the evaporation of water; the second step of 5.85% from 110–800 °C, resulting from the condensation of the silanol groups.^56,57^SiEp also exhibited two decomposition stages at 1.34% from 25–110 °C and 10.8% from 200 to 800 °C due to the desorption of water and the degradation of organic moieties grafted to silica. Therefore, the two distinct weight loss steps of SiPy represent a loss of 4.13% in the 25–100 °C range and 12.4% in the interval of 270–800 °C, which were attributed to the vaporization of water and degradation of organic compounds.

### 3.3. Adsorption studies

#### 3.3.1. Effect of pH

**Fig. 5:**
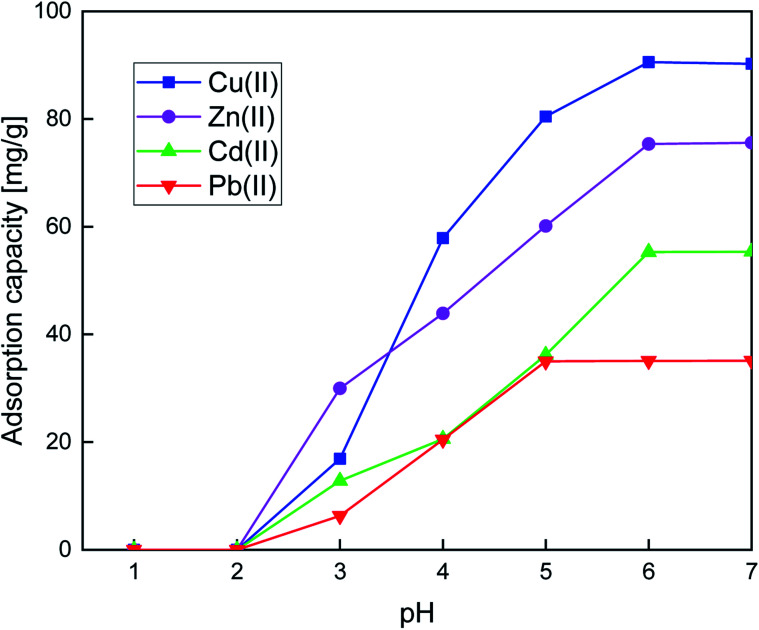
The effect of pH on the adsorption capacity of SiPy toward Cu(ii), Cd(ii), Cd(ii), and Pb(ii). Adsorption condition: *V* = 10 mL, *m* = 10 mg of adsorbent and optimum concentrations = 180.66 10^−3^, 140.10 10^−3^, 99.70 10^−3^, and 95 63 10^−3^ g L^−1^ for Cu(ii), Zn(ii), Cd(ii), and Pb(ii) respectively, *t* = 25 min at 25 °C.

The adsorption of heavy metal ions depends on the pH of the solution, which is a dominant parameter affecting the adsorption. In this investigation, the effect of pH on the adsorption experiments was performed on SiPy over the range of 1.0–7.0 with optimal initial concentration (Fig. 5). Herein, the adsorption amount of the SiPy adsorbent increases with increasing pH. According to Fig. 5, the optimal pH values for the sorption of heavy metal ions by SiPy are about 6.0 and 7.0. At low pH, the adsorption is mainly due to a large number of H_3_O^+^ in the suspension, which are available to protonate the pyridine unit and oxygen atoms and renders the surface of SiPy positively charged, which had a repulsive force that hampered the removal of metal ions. At pH ≥8, SiPy adsorption efficiency remains almost constant, indicating the formation of metal hydroxide precipitates for Cu(ii), Zn(ii), Cd(ii), and Pb(ii) ions.

#### 3.3.2. Effect of contact time and adsorption mechanism

**Fig. 6:**
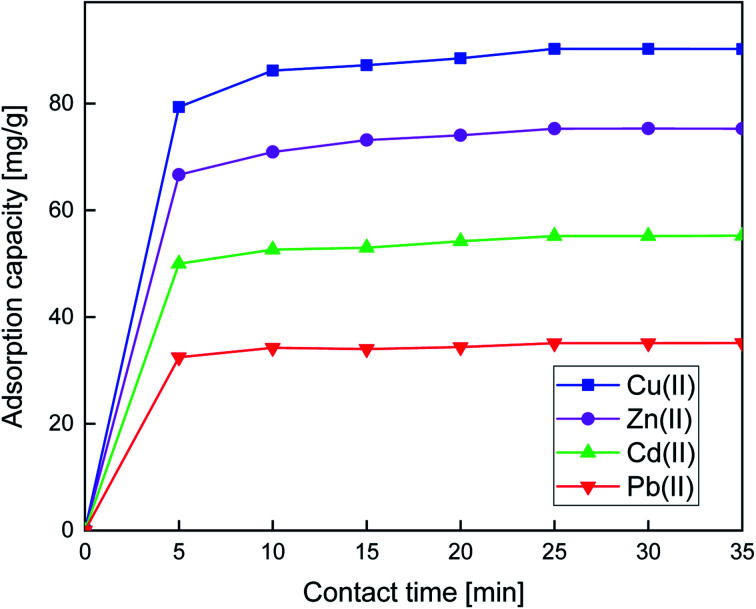
Effect of contact time on the adsorption capacity of Cu(ii), Zn(ii), Cd(ii), and Pb(ii). Adsorption conditions: *V* = 10 mL, *m* = 10 mg of adsorbent, pH = 6, and optimum concentrations: 180.66 10^−3^, 140.10 10^−3^, 99.70 10^−3^, and 95 63 10^−3^ g L^−1^ for Cu(ii), Zn(ii), Cd(ii), and Pb(ii), respectively, at 25 °C.

The study of the impact of the contact time is very important for the investigation of metal ions removal. For this purpose, the time of adsorption of Cu(ii), Cd(ii), Cd(ii), and Pb(ii) was varied from 5 to 35 min. From Fig. 6, the adsorption capacity increased rapidly during the first 15 min and then reached equilibrium; this indicates the high dispersibility of the organic group receptor in the aqueous phase, and likely stems from the rapid interaction between the individual metal ions and the active sites of the adsorbent.

The potential mechanisms controlling the sorption of Cu(ii), Zn(ii), Cd(ii), and Pb(ii) by SiPy were studied using first, second-order, and intra-particle diffusion models. The linear form of the three models can be calculated as eqn (2)–(4).^58,59^

Pseudo-first-order model:

2 ln(*q*_e_ − *q*_*t*_) = ln *q*_e_ − *k*_1_*t*

where *q*_e_ (mg g^−1^) and *q*_*t*_ (mg g^−1^) are the amounts of adsorbate at equilibrium and at time *t* (s), respectively, and *K*_1_ (min^−1^) is the rate constant of the first-order adsorption.

Pseudo-second-order model:

3 *t*/*q*_*t*_ = 1/*K*_2_*q*_e_^2^ + *t*/*q*_e_

where *K*_2_ (g (mg min^−1^)^−1^) is the second order rate constant of adsorption.

Intra-particle diffusion model:

4 *q*_*t*_ = *k*_pi_*t*^1/2^ + *C*_i_

where *k*_pi_ (mg g^−1^ min^−1/2^) is the intra-particle diffusion rate constant, and constant *C*_i_ indicates the thickness of the boundary layer.

The Cu(ii), Zn(ii), Cd(ii), and Pb(ii) experimental adsorption rate data were fitted to the three kinetic models. All the kinetic data of adsorption onto SiPy, calculated from the related linear fitting curves, are shown in Fig. S1 and S2.† The corresponding parameters by the pseudo-second-order kinetic model are listed in Fig. 6 and Table 1. The values *R* estimated from the pseudo-second-order kinetic model were significantly higher than those obtained from the pseudo-first-order kinetic model. The results suggested a better fitting of the pseudo-second-order, assuming that the adsorption was a chemisorption process.

**Table:** Kinetic model data of Cu(ii), Zn(ii), Cd(ii), and Pb(ii) adsorption

Parameters	Metal ions			
Experimental	Cu(ii)	Zn(ii)	Cd(ii)	Pb(ii)
*q*_e(exp)_ (mg g^−1^)	90.25	75.38	55.23	35.12

**Pseudo-first order**				
*q*_e (_mg g^−1^)	27.234	16.09	8.309	2.872
*k*_1_ (g mg^−1^ min^−1^)	0.135	0.127	0.100	0.070
*R* ^2^	0.8891	0.9963	0.9481	0.6647

**Pseudo-second order**				
*q*_e (_mg g^−1^)	92.592	77.519	56.497	35.714
*k*_2_ (g mg^−1^ min^−1^)	0.013	0.016	0.023	0.050
*R* ^2^	0.9999	0.9999	0.9998	0.9965

Then, it is important to further study the intra-particle diffusion model, which has been widely applied to provide the rate-controlling steps affecting the adsorption mechanism.^60,61^ Fig. S3a–d† shows the plot of *q*_e_*vs. t*^1/2^ accompanied by two distinct stages with different line slopes. Lines do not pass through the origin, indicating that the mechanism is complex and that intra-particle diffusion is not the only step controlling the adsorption process but that two steps occurred during the adsorption process. In this case, both external and internal diffusion can be involved in the adsorption mechanism. The first step corresponded to the instantaneous adsorption or external surface adsorption and the second to the progressive adsorption or intra-particle diffusion stage. Table 2 shows that the diffusion rate constants decreased following the order *k*_p1_ > *k*_p2_. The high slopes of the first step indicate that the removal rate of Cu(ii), Zn(ii), Cd(ii), and Pb(ii) ions is higher at the beginning of the process due to the availability of a large number of active sites on the adsorbent surface. The lower slopes of the second parts are due to the decrease in the concentration gradient, which makes the diffusion of Cu(ii), Zn(ii), Cd(ii), and Pb(ii) ions into the mesopores of the adsorbent slower, thus leading to a low removal rate. As the data showed, intra-particle diffusion was the real rate-controlling process of the adsorption process.

**Table:** Intra-particle diffusion parameters for Cu(ii), Zn(ii), Cd(ii), and Pb(ii) adsorption onto SiPy

Metal ions	*K*_p1_	*C*_1_	*R* ^2^	*K*_p2_	*C*_2_	*R* ^2^
Cu(ii)	3.941	71.755	0.875	−0.011	90.3	0.728
Zn(ii)	3.367	59.635	0.960	0.011	75.251	0.861
Cd(ii)	1.779	46.345	0.935	0.054	54.906	0.980
Pb(ii)	0.777	31.097	0.716	0.01	35.054	0.728

#### 3.3.3. Influence of initial concentration and adsorption isotherms

**Fig. 7:**
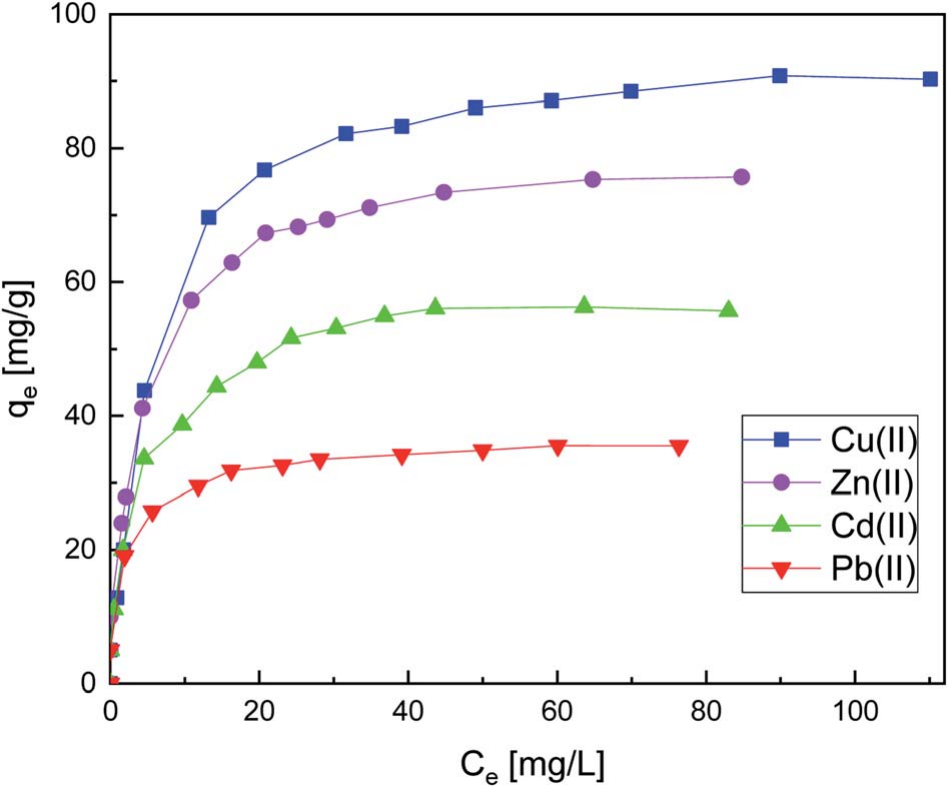
Effect of concentration on metal ion adsorption onto SiPy adsorption. Adsorption conditions: *m* = 10 mg, *V* = 10 mL, [Mn(ii)] = 10 to 300 × 10^−3^ g L^−1^, pH = 6, time = 25 min at 25 °C.

The impact of varying concentration of metal ions has been evaluated under optimal conditions. Fig. 7 indicates that the adsorption efficiency of all the metal ions increased with the increase in the initial concentration of Cu(ii), Zn(ii), Cd(ii), and Pb(ii). These very high adsorption capacities can be justified by the available adsorption sites on the surface of the adsorbents, which are easily occupied by the metal ions. But the number of active sites on the SiPy surface is limited when they are fully occupied. Thus, saturated adsorption would be achieved at that time.^62^ The equilibrium adsorption of the metal ions on SiPy was in the order Cu(ii) > Zn(ii) > Cd(ii) > Pb(ii) (Fig. 7).

The adsorption mechanism of metal ions and the SiPy adsorbent was investigated by Langmuir, Freundlich, Dubinin–Radushkevich (D–R), and Temkin isotherm models,^63,64^ which are recalled below:

Langmuir model:

5 *C*_e_/*q*_e_ = *C*_e_/*q*_m_ + 1/*qK*_L_

where *q*_e_ (mg g^−1^) and *q* (mg g^−1^) represent the equilibrium and the saturated adsorption capacity, respectively. *C*_e_ (mg g^−1^) is the concentration of metal ions in solution at equilibrium, *q*_m_ (mg g^−1^) is the theoretical saturation adsorption capacity, and *K*_L_ (L mg^−1^) is the equilibrium Langmuir constant.

Freundlich model:

6 *q*_e_ = *K*_F_*C*_e_^1/*n*^

Where *K*_F_ is the Freundlich constant and *n* is the value used to suggest the heterogeneity of the interface.

Dubinin–Radushkevich (D–R) model:

7 ln(*q*_e_) = ln(*q*_m_) − *βε*^2^

where *β* (mol J^−1^)^2^ is a constant related to the mean free energy *E* of adsorption (*E* = (2*β*)^−0.5^), and *ε* (J mol^−1^) is the Polanyi potential related to the equilibrium concentration (*ε* = *RT* ln(1 + 1/*C*_e_)).

Temkin model:

8 *q*_e_ = *RT*/*b*_*t*_ ln *A*_*t*_ + *RT*/*b*_*t*_ ln *C*_e_

where *b*_*t*_ (J mol^−1^) and *A*_*t*_ (L mg^−1^) are the Temkin isotherm constant, *T* defines the Kelvin temperature (K), and *R* (8.314 J mol^−1^ K^−1^) defines the universal gas constant.

The plots of the linear fitting curves of the Langmuir, Freundlich, D–R, and Temkin isotherm mod-els for the adsorption of Cu(ii) Zn(ii), Cd(ii), and Pb(ii) onto the surface of SiPy are presented in Fig. S4–S7.† The experimental data and the fitted curves for the single-metal system are shown in Fig. 7 with the corresponding parameters listed in Table 3.

Parameters for the Langmuir, Freundlich, Dubinin–Radushkevich (D–R), and Temkin models of Cu(ii) sorption

**Table:** 

	Langmuir model			Freundlich model		
	*q*_e_ (mg g^−1^)	*K*_L_ (L mg^−1^)	*R* ^2^	*K*_F_ (mg g^−1^)	*N*	*R* ^2^
Cu(ii)	95.602	0.178	0.999	18.250	2.527	0.898
Zn(ii)	79.365	0.250	0.999	24.574	3.354	0.912
Cd(ii)	58.823	0.288	0.998	17.131	3.147	0.912
Pb(ii)	36.63	0.400	0.999	18.840	6.146	0.924

**Table:** 

	Dubinin–Radushkevich (D–R) model			Temkin model		
	*β* (mol^2^ kJ^−2^)	*E* (kJ mol^−1^)	*R* ^2^	*A*_*t*_ (L mg^−1^)	*b*_*t*_ (J mol^−1^)	*R* ^2^
Cu(ii)	6.02 × 10^−7^	912.87	0.838	2.601	142.06	0.965
Zn(ii)	7.83 × 10^−7^	799.10	0.892	4.048	178.17	0.965
Cd(ii)	2.01 × 10^−7^	1581.13	0.782	4.949	253.93	0.975
Pb(ii)	5.28 × 10^−7^	973.12	0.848	10.85	555.63	0.959

It is obvious that the *R*^2^ values are higher for the Langmuir model, thus confirming that the adsorption process belongs to monolayer adsorption. The maximum adsorption capacity is found for Cu(ii) with 95.60 mg g^−1^, a high value that shows that the SiPy adsorbent could be used potentially for the adsorption of Cu(ii) from industrial wastewater and river waters.

#### 3.3.4. Adsorption thermodynamics

The effect of temperature on sorption experiments was also investigated. Gibbs free energy Δ*G*° (kJ mol^−1^), enthalpy Δ*H*° (kJ mol^−1^), and entropy Δ*S*° (J mol^−1^ K^−1^) were obtained from eqn (9)–(11).^65,66^

9 *K*_d_ = (*C*_0_ − *C*_e_)/*C*_e_

10 ln *K*_d_ = Δ*S*°/*R* − Δ*H*°/*RT*

11 Δ*G*° = Δ*H*° − *T*Δ*S*°

*R* (8.314 J mol^−1^ K^−1^), *T* (K), and *K*_d_ are the universal gas constant, absolute temperature, and distribution coefficient, respectively.

**Fig. 8:**
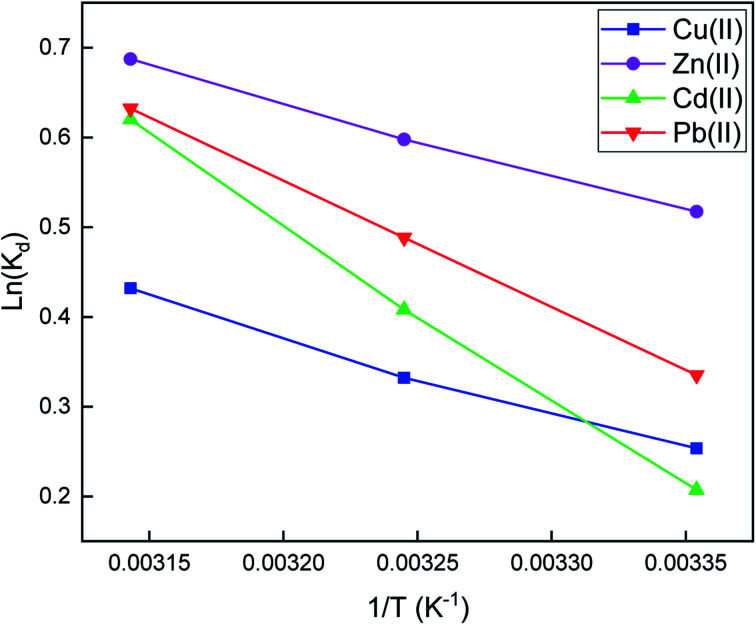
Effect of temperature for the adsorption of metal ions onto the SiPy adsorbent.

All thermodynamic parameters are listed in Table 4. The negative values of Δ*G*° suggest that the adsorption process of SiPy for Cu(ii), Zn(ii), Cd(ii), and Pb(ii) is thermodynamically favorable and spontaneous. Meanwhile, the positive value of Δ*H*° indicates that the adsorption process has an endothermic nature. On the other hand, the obtained negative values of Δ*S*° points out an increase in the randomness and disorder at the surface of the adsorbent Fig. 8.

**Table:** Thermodynamic parameters

Metal	Δ*H*° (kJ mol^−1^)	Δ*S*^°^ (J K^−1^ mol^−1^)	*T* ± 1 °C	Δ*G*^°^ (kJ mol^−1^)
Cu(ii)	8.1364	29.163	25	−0.558
			35	−0.850
			45	−1.141
Zn(ii)	6.6921	26.725	25	−1.276
			35	−1.810
			45	−1.543
Cd(ii)	16.2538	56.2	25	−0.502
			35	−1.064
			45	−1.626
Pb(ii)	11.706	42.06	25	−0.834
			35	−1.254
			45	−1.675

#### 3.3.5. Adsorption selectivity for Cu(ii)

**Fig. 9:**
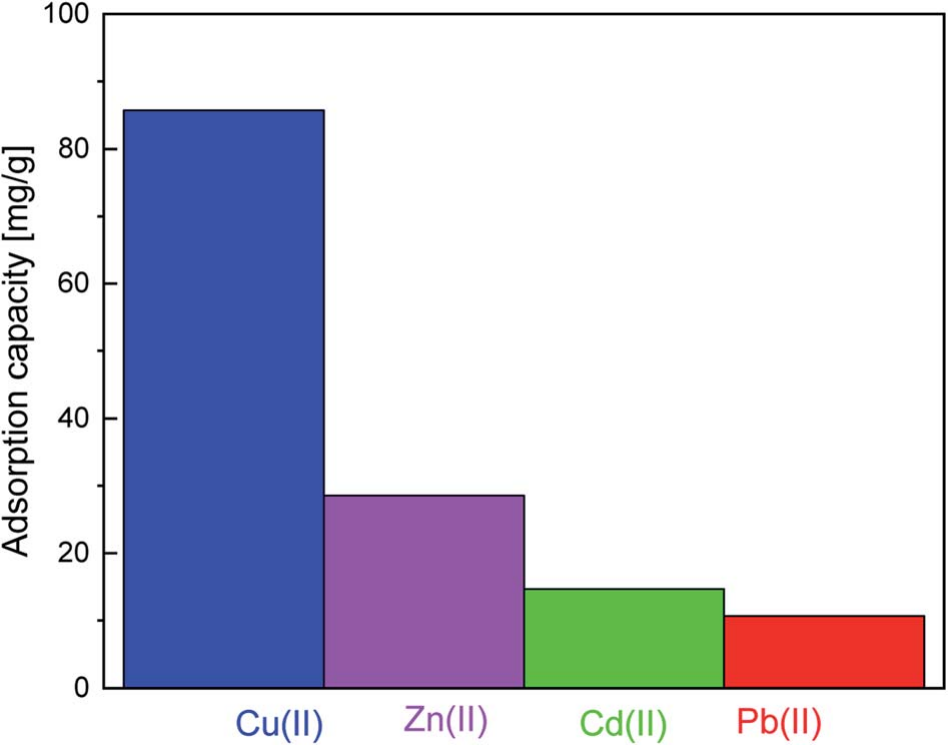
Metal ion selectivity effect for SiPy.

The adsorption selectivity of SiPy was examined using aqueous solutions containing Cu(ii), Zn(ii), Cd(ii), and Pb(ii) with optimum concentration of each metal ion (180.66 10^−3^, 140.10 10^−3^, 99.70 10^−3^, and 95 63 10^−3^ g L^−1^ for Cu(ii), Zn(ii), Cd(ii), and Pb(ii), respectively). According to Fig. 9, SiPy shows excellent adsorption selectivity for Cu(ii) ions. Furthermore, given this selectivity, our adsorbent could be tested for its applicability in the removal of Cu(ii) from river waters, which is discussed in the next section.

#### 3.3.6. Desorption and recycling

As adsorbents, regeneration is of significant importance for any application that guarantees economic perspectives and durability of operation in practice. The adsorbent was subjected to five cycles of Cu(ii) adsorption using HCl (2 mol L^−1^) as the eluent. After five regeneration cycles, the adsorption efficiency of the adsorbent for Cu(ii) can still reach 93.81% (Table 5). This result confirms the stability of the prepared SiPyand establishes the feasibility of the high regenerative capacity of the adsorbent.

**Table:** Reusability and recycling of the SiPy adsorbent toward Cu(ii) in the adsorption–desorption cycles

Cycle	*q*_e_ (mg g^−1^) of Cu(ii) adsorbed on SiPy
1	90.25
2	89.45
3	88.02
4	86.13
5	84.67

#### 3.3.7. Application in real water treatment

In order to test its feasibility in a real application, an adsorption experiment was carried out on real water samples originating from (i) Ghis river (located next to Al Hoceima) where: pH = 7.7, total dissolved solids (TDS) = 1297 mg L^−1^ and conductivity *σ* = 1733 μS cm^−1^; (ii) Touissit–Boubekker river (in the Jerada–Oujda region) where: pH = 7.1, TDS = 2031 mg L^−1^ and *σ* = 2301 μS cm^−1^. The adsorption efficiency of SiPy (10 mg) was investigated under optimal conditions by the batch method using 10 mL of the river water. The percentage removal efficiency of Cu(ii) ion was found to be high, up to 92% and 94% from Ghis and Touissit–Boubekker rivers, respectively, for SiPy (Table 6). Thus, SiPy is not only an economic adsorbent but it is also potentially feasible to be transformed to a high value-added product, *i.e.*, a material for larger industrial application for extracting Cu(ii) from wastewater.

**Table:** Removal of copper from real wastewater samples using SiPy

Rivers	Added Cu(ii) (mg L^−1^)	Adsorption capacity (mg g^−1^)	Percentage of adsorption efficiency (%)
Ghis	10	9.26	92.60
Touissit–Boubekker	10	9.45	94.50

#### 3.3.8. Comparison with similar adsorbents

Table 7 shows the comparison of the adsorption capacity of SiPy for Cu(ii) with other literature adsorbents. It is clear that SiPy has a higher adsorption efficiency for Cu(ii) ion (90.25 mg g^−1^).

**Table:** Comparison of the maximum adsorption capacities of Cu(ii) by different adsorbents reported in the literature

Silica gel-ligand	Metal ion (mg g^−1^)	Reference
Pyridin-2-ylmethanol	90.25	This work
Porphyrin	19.08	67
*N*-Propyl-2-pyridylimine	35.63	68
Methyl methacrylate	41.36	69
Dithiocarbamate	25.00	70
(*E*)-4-(Furan-2-ylmethyleneamino) phenol	36.20	71
(*E*)-2-(Furan-2-ylmethyleneamino) phenol	79.36	71
3-Hydroxysalicylaldiminepropyltriethoxy-silane	5.72	72
Furan ketone enol	31.82	73
3-Amino-1,2-propanediol	31.18	74
Commercial Lewatit (L-207)	68.09	75
Bis(pyrazole)butane	20.24	76

### 3.4. Adsorption mechanism

#### 3.4.1. FTIR technique

**Fig. 10:**
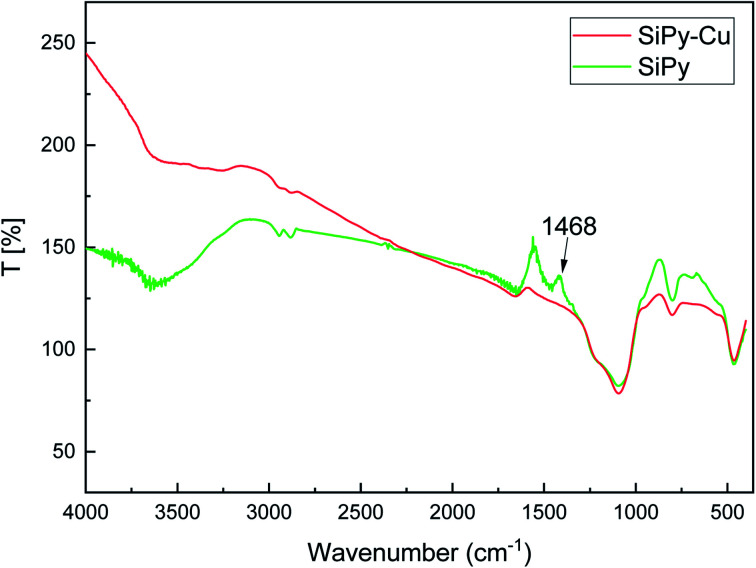
FTIR spectra of SiPy before and after Cu(ii) ion adsorption.

The organic fraction of the surface of the material plays an important role in the removal of metal ions. The FTIR spectra of SiPy before and after the adsorption of Cu(ii) indicate that the absorption peak at 1462 cm^−1^ belonging to the N=C group disappeared after the adsorption of Cu(ii) (Fig. 10).^77^ The results demonstrate that the N=C group exhibits marked binding of Cu(ii).

#### 3.4.2. Theoretical investigations

Theoretical investigation was undertaken to get insights into the mechanism of adsorption of metal ions with SiPy. The following theoretical methods were used: DFT, QTAIM, LOL, NCI, and dual descriptor.^78^

**Fig. 11:**
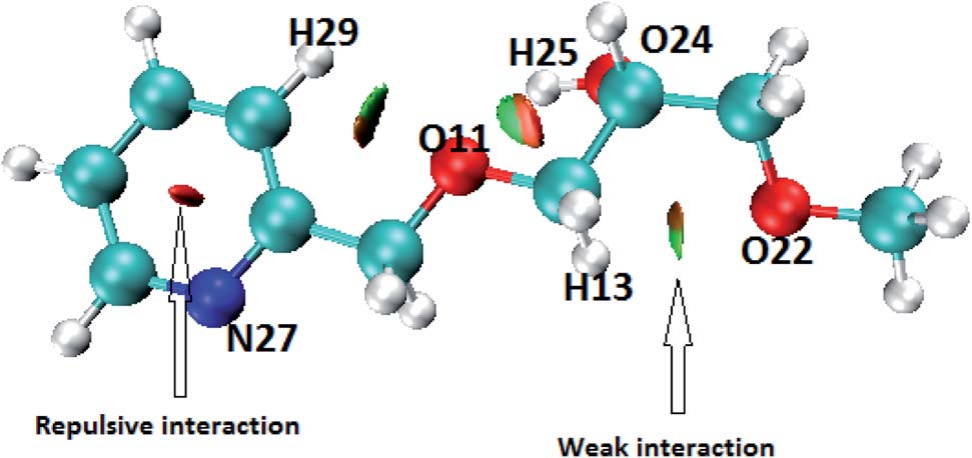
NCI isosurface for SiPy. Weak interactions are shown in green, repulsive interactions in red, and strong interactions in blue.

Weak interactions were found between oxygen and hydrogen atoms (O_11_⋯H_29_), (O_11_⋯H_25_), (O_22_⋯H_13_) as well as a repulsive interaction within the pyridine ring (Fig. 11). Since nitrogen N_27_ and O_24_ do not interact, we can deduce that these atoms may be involved in coordination with metal ions to give the SiPy–metal hybrid. Oxygen O_11_ only makes weak interaction with the hydrogen atoms so it can be still be bound to the metals.

**Fig. 12:**
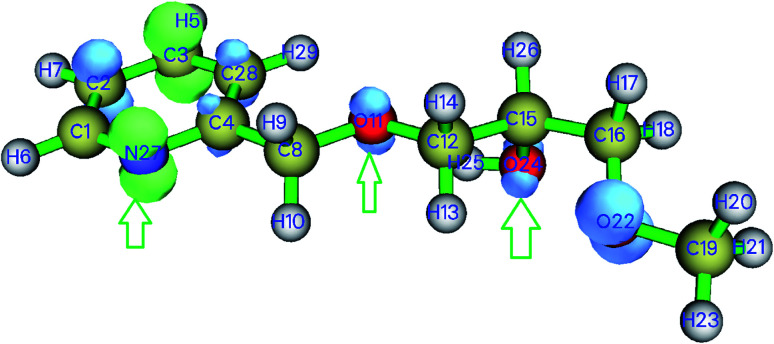
Dual descriptors isosurface for SiPy.

**Fig. 13:**
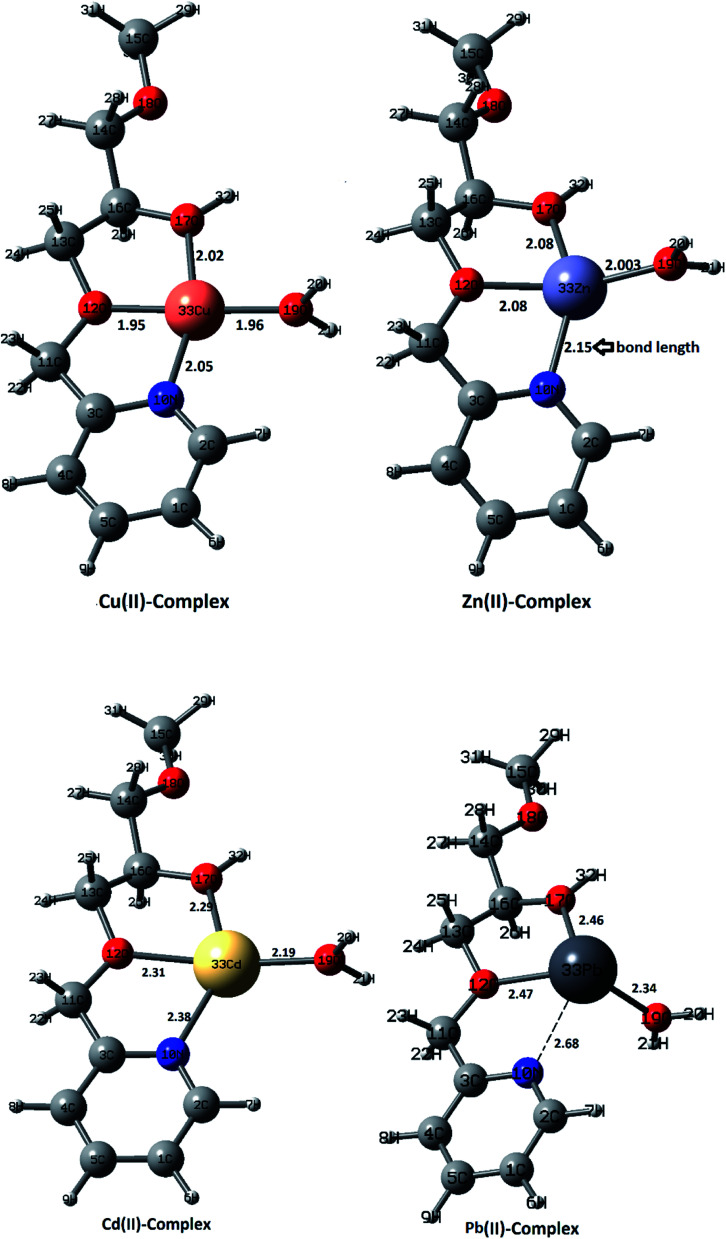
Optimized structures of ligand–metal ion–H_2_O complexes, and bond lengths in Å.

Dual descriptors are a useful function used to reveal the reactive sites. Fig. 12 shows that N_27_, O_11_, O_24_ are available for coordination with metal ions. Actually, NCI and dual descriptors confirm that these three are responsible for forming the SiPy–metal hybrid. An optimization by the DFT(B3LYP) method afforded all possible structures of (Cu(ii)–SiPy, Zn(ii)–SiPy, Cd(ii)–SiPy, and Pb(ii)–SiPy (Fig. 13)). The bond lengths between the metal and the neighboring atoms as well as the coordination type is also given. The optimized (M(ii)–SiPy) structures form stable complexes but these complexes do not have the same stability, given the bond lengths and the type of coordination (Fig. 13). For instance, the bond lengths of Cu(ii)–SiPy (Cu_33_–O_12_ = 1.95 Å) are smaller compared to those for Zn(ii)–SiPy (Zn_33_–O_12_ = 2.08 Å) and even smaller for Cd(ii)–SiPy Cd–O_12_ = 2.31 Å) and Pb(ii)–SiPy (Pb_33_–O_12_ = 2.47 Å).

A tridentate coordination mode is noticed for (Cu(ii), Zn(ii), Cd(ii))–SiPy, whereas another coordination site is occupied by a water molecule. A bidentate coordination mode is however noticed for Pb(ii)–SiPy, which supports the stability order Cu(ii)–SiPy > Zn(ii)–SiPy > Cd(ii)–SiPy > Pb(ii)–SiPy.

In order to better understand metal coordination, we have used the NBO (natural bond orbital)^79^ method, which allows to highlight the adsorption selectivity of the metal ions toward the ligand, evaluate the interactions at the complex level (M(ii)–N_10_, M(ii)–O_12_, M(ii)–O_17_) with the use of *E*^(2)^ (second-order stabilization energy) and EC (electronic configurations).

The values of *E*^(2)^ are useful to indicate the binding strength, which is found to be higher for the Cu(ii) complex with a charge transfer LP(O_12_) → LP*(Cu(ii)) of 16.63 kcal mol^−1^ compared to LP(O12) → LP*(Zn(ii)) = 16.11 kcal mol^−1^ and LP(O12) → LP*(Zn(ii)) = 6.03 kcal mol^−1^ for Zn(ii) (Table 8). For the Pb(ii)–SiPy complex, a lower *E*^(2)^ value was found, indicating a low interaction strength of Cu(ii)–O10. Thus, the computational investigation completely replicates the experimental selectivity (Fig. 9).^80^

**Table:** Calculations for the metal complexes using NBO analysis

	EC of M(ii), N_10_, O12, and O_17_	*E* ^(2)^ energy (kcal mol^−1^)		
		LP(N_10_) → LP*(M)	LP(O_12_) → LP*(M)	LP(O_17_) → LP*(M)
Cu(ii) complex	Cu_33_[core]4S(0.26)3d(9.28)4p(0.20)	21.29	16.63	11.84
	N_10_[core]2S(1.38)2p(4.23)3p(0.02)			
	O_12_[core]2S(1.64)2p(5.00)3p(0.01)			
	O_17_[core]2S(1.68)2p(5.14)3p(0.01)			
Zn(ii) complex	Zn_33_[core]4S(0.24)3d(9.98)4p(0.20)	16.61	16.11	2.34
	N_10_[core]2S(1.40)2p(4.29)3p(0.02)			
	O_12_[core]2S(1.64)2p(5.01)3p(0.01)			
	O_17_[core]2S(1.69)2p(5.18)3p(0.01)			
Cd(ii) complex	Cd_33_[core]5S(0.22)4d(9.99)5p(0.12)	5.05	6.03	0.28
	N_10_[core]2S(1.41)2p(4.27)3p(0.02)			
	O_12_[core]2S(1.65)2p(4.99)3p(0.01)			
	O_17_[core]2S(1.70)2p(5.18)3p(0.01)			
Pb(ii) complex	Pb_33_[core]6S(1.95)6p(0.32)	18.20	4.67	1.16
	N_10_[core]2S(1.41)2p(4.23)3p(0.02)			
	O_12_[core]2S(1.67)2p(4.99)3p(0.01)			
	O_17_[core]2S(1.71)2p(5.17)3p(0.01)			

All the interatomic areas have positive ∇^2^*ρ*_cp_ values, and a low value of the electron density, *ρ*_cp_ ≈ 0 (Table 9), which shows that the interatomic surface cps has identified non-covalent bonds. Thus, the involved bond in coordination is either ionic or of van der Waals type. However, the value of the electron density of the Cu(ii) complex is *ρ*_cp_ = 0.08 (Cu_33_–O_10_), which is higher compared to the ones found for the Zn(ii) complex with *ρ*_cp_=0.05 (Zn_33_–O_10_), as well as for the Cd(ii)complex with *ρ*_cp_=0.02 (Cd–O_10_) and the Pb(ii) complex. Thus, the stability of the four complexes follows the order Cu(ii) > Zn(ii) > Cd(ii) > Pb(ii).

**Table:** Calculation of the electron density *ρ*_cp_ and the Laplacian ∇^2^*ρ*_cp_ (a.u) of cps (M–O, M–N) found by QTAIM

Entry	Cp	Bonding region	*ρ*_cp_	∇^2^*ρ*_cp_
Cu(ii)-complex	63	Cu_33_–N_10_	0.07	0.29
	59	Cu_33_–O_10_	0.08	0.45
	62	Cu_33_–O_17_	0.06	0.33
	68	Cu_33_–O_19_	0.07	0.43
Zn(ii)-complex	63	Zn_33_–N_10_	0.05	0.23
	59	Zn_33_–O_10_	0.05	0.31
	62	Zn_33_–O_17_	0.05	0.30
	68	Zn_33_–O_19_	0.06	0.40
Cd(ii)-complex	63	Cd_33_–N_10_	0.04	0.21
	61	Cd_33_–O_10_	0.02	0.15
	62	Cd_33_–O_17_	0.04	0.27
	69	Cd_33_–O_19_	0.06	0.35
Pb(ii)-complex	41	Pb_33_–N_10_	0.03	0.10
	46	Pb_33_–O_10_	0.04	0.16
	38	Pb_33_–O_17_	0.04	0.17
	36	Pb_33_–O_19_	0.05	0.22

**Fig. 14:**
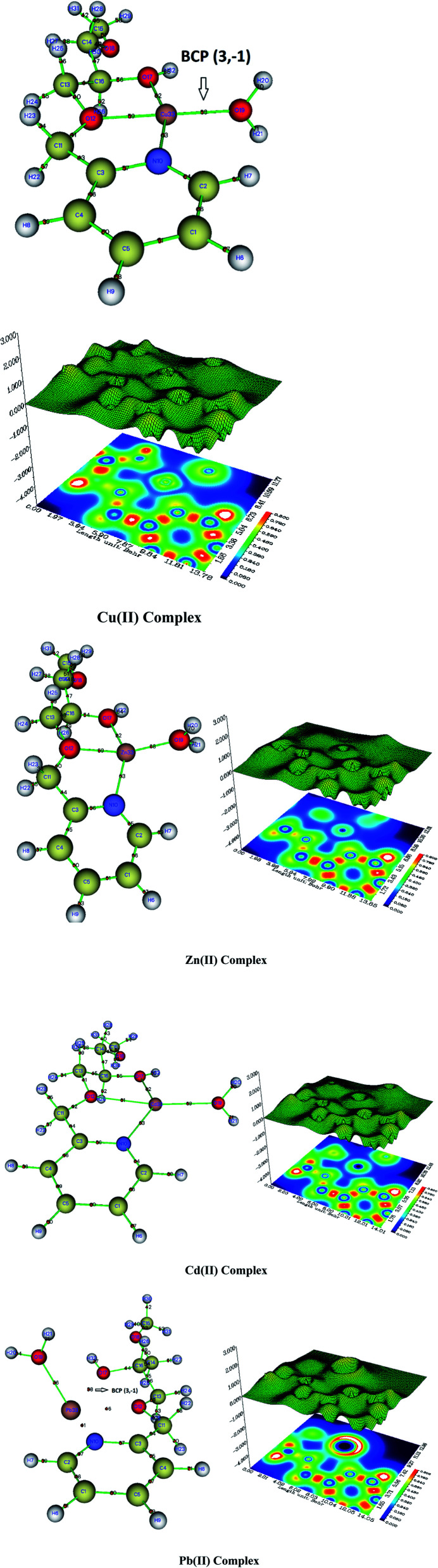
Localized orbital locator (LOL) topology analysis of M(ii) complexes, and bond critical point (Bcp) associated with the formation of M–(O, N) bond, contour map, in plane (N_10_, O_12_, M_33_).

The structures of the complexes shown in Fig. 14 show the BCP point for the (M(ii)33–N10, M(ii)33–O12, M(ii)33–O17, and M(ii)33–O19), which confirms the stability of the complexes. In the contour map, the blue color represents a low electron density region, whereas the red color shows a high electron density. The purple color means the absence of electron density (valence electrons). Such a color is not found for the Cu(ii) complex, whereas a small area is detected for the Zn(ii) complex. However, a larger area is found for the Cd(ii) complex, and the Pd(ii) complex has a much larger purple area. Thus, the stability of our four complexes is confirmed as follows: Cu(ii) > Zn(ii) > Cd(ii) > Pb(ii).

## 5. Conclusion

In conclusion, a new simple and low-cost method for the highly efficient cleanup of heavy metal ions in water has been presented, using silica organically modified with pyridine, which has been synthesized and fully characterized. The maximum sorption capacity after reaching the equilibrium (30 min) was 90.25, 75.38, 55.23, and 35.12 mg g^−1^ for Cu(ii), Zn(ii), Cd(ii), and Pb(ii) respectively, with an optimal sorption pH of 7. The adsorption kinetics process is dominated by the pseudo-second-order model, which indicates a homogeneous character. The adsorption properties indicated monolayer adsorption, chemisorption binding mechanism, and an endothermic and spontaneous process. The SiPy adsorbent was able to maintain 93.81% of Cu adsorption capacity even after five cycles of adsorption and desorption experiments. A computational study suggested that the removal of the metal ions is the synergistic effect of C=N, R–O–R, and R–OH groups functionalized onto the SiPy material. The C=N group plays a major function during the complexation of metal ions. Furthermore, the computational investigation confirmed the affinity of our hybrid material for Cu(ii). The results suggested that the SiPy adsorbent displays great advantages of high adsorption capacity toward Cu(ii), rapid response, high selectivity, and good reusability, which makes it as a good candidate for wastewater treatment applications.

## Conflicts of interest

The authors declare that they have no known competing financial interests or personal relationships that could have appeared to influence the work reported in this paper.

## Supplementary Material

RA-012-D1RA06640D-s001
