# Supplementary material for: Kinetics, thermodynamics, equilibrium, surface modelling, and atomic absorption analysis of selective Cu(ii) removal from aqueous solutions and rivers water using silica-2-(pyridin-2-ylmethoxy)ethan-1-ol hybrid material
Source: RSC Adv. 2021 Dec 22;12(1):611–25. doi: 10.1039/d1ra06640d (PMC8978821; doi:10.1039/d1ra06640d)
Supplement: RA-012-D1RA06640D-s001 [file RA-012-D1RA06640D-s001.pdf]

Electronic Supplementary Material (ESI) for RSC Advances.
This journal is © The Royal Society of Chemistry 2021

Supporting Information

Kinetics, thermodynamics, equilibrium, surface modelling and atomic absorption analysis of selective Cu(II) removal from aqueous solutions and rivers water using silica-2-(pyridin-2-ylmethoxy)ethan-1-ol hybrid material

Said Tighadouini[1]*, Smaail Radi[2]*, Othmane Roby[1], Imad Hammoudan[1], Rafik Saddik[1], Yann Garcia[3]*, Zainab M. Almarhoon[4], Yahia N. Mabkhot[5]*

[1] Laboratory of Organic Synthesis, Extraction and Valorization, Faculty of Sciences Ain Chock, Hassan II University, BP: 5366, Casablanca, Morocco. (Emails: tighadouinis@gmail.com (S.T); othmaneroby1@gmail.com (O.R); hammoudanimad18@gmail.com (I.H); rafik.saddik@gmail.com (R.S)).

[2] Laboratory of applied Chemistry and Environment (LCAE), Faculty of Sciences, University Mohamed Premier, 60000 Oujda, Morocco. (Email: s.radi@ump.ac.ma).

[3] Institute of Condensed Matter and Nanosciences, Molecular Chemistry, Materials and Catalysis (IMCN/MOST), Université catholique de Louvain, Place Louis Pasteur 1, 1348 Louvain-la-Neuve, Belgium (Email: yann.garcia@uclouvain.be).

[4] Department of Chemistry, College of Science, King Saud University, P.O. Box 2455, Riyadh 11451, Saudi Arabia (E-mail: zalmarhoon@ksu.edu.sa).

[4] Department of Pharmaceutical Chemistry, College of Pharmacy, King Khalid University, Abha, P.O. Box 960, Postal code 61421, Saudi Arabia (Email: ygaber@kku.edu.sa).

*Corresponding authors: tighadouinis@gmail.com (S. Tighadouini), s.radi@ump.ac.ma (S. Radi), ygaber@kku.edu.sa (Y.N. Mabkhot), yann.garcia@uclouvain.be (Y. Garcia)

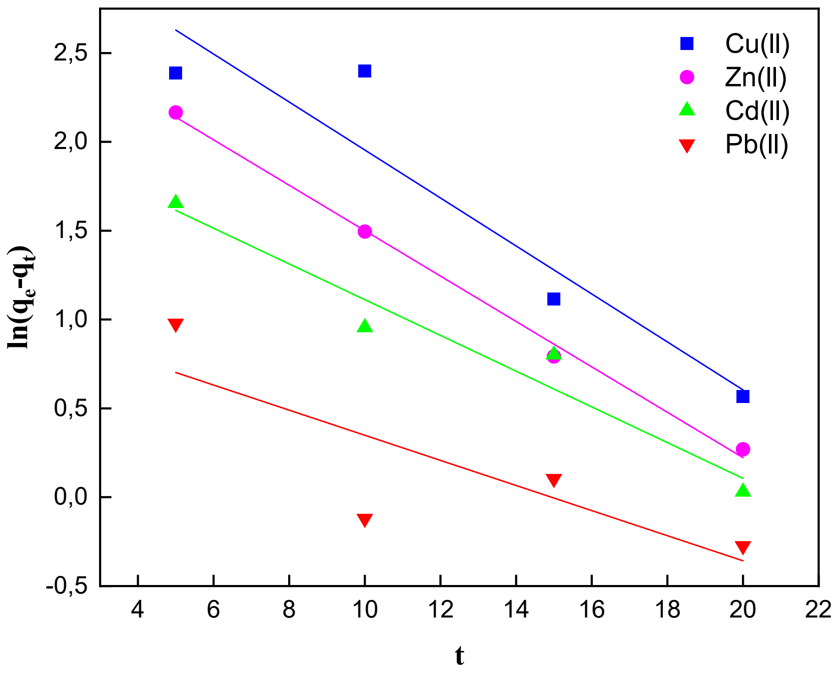

Fig. S1 Kinetic curves of Cu(II), Zn(II), Cd(II) and Pb(II) adsorption rate onto **SiPy** fitted by pseudo-first-order. Adsorption conditions: V = 10 mL, m = 10 mg of adsorbent, pH = 6, and optimum concentrations: 180.66, 140.10, 99.70 and 95,63.10^{-3} mg L^{-1} for Cu(II), Zn(II), Cd(II) and Pb(II) respectively at 25 °C.

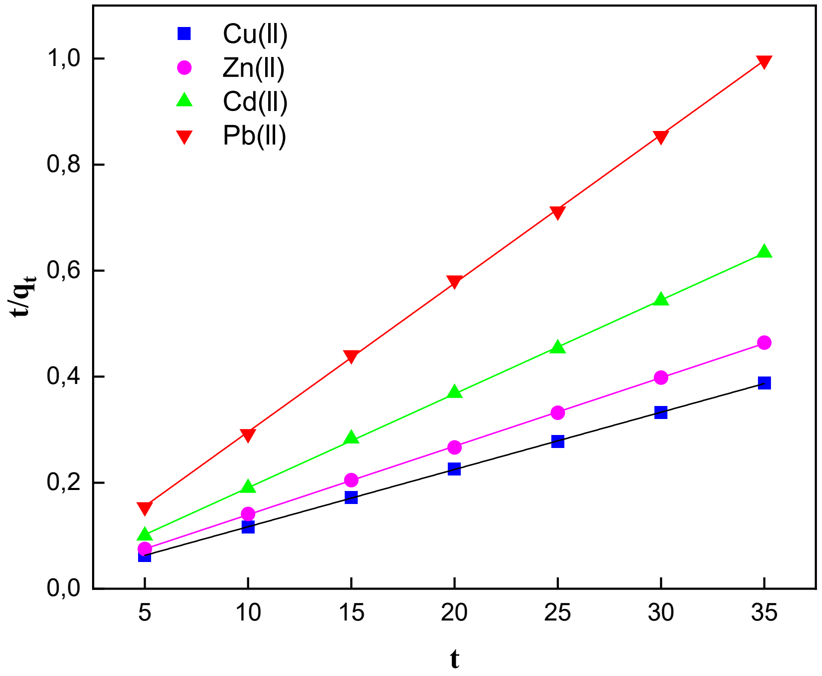

Fig. S2 Kinetic curves of Cu(II), Zn(II), Cd(II) and Pb(II) adsorption rate onto **SiPy** fitted by pseudo-second-order. Adsorption conditions: V = 10 mL, m = 10 mg of adsorbent, pH = 6, and optimum concentrations: 180.66, 140.10, 99.70 and 95,63.10^{-3} mg L^{-1} for Cu(II), Zn(II), Cd(II) and Pb(II) respectively at 25 °C.

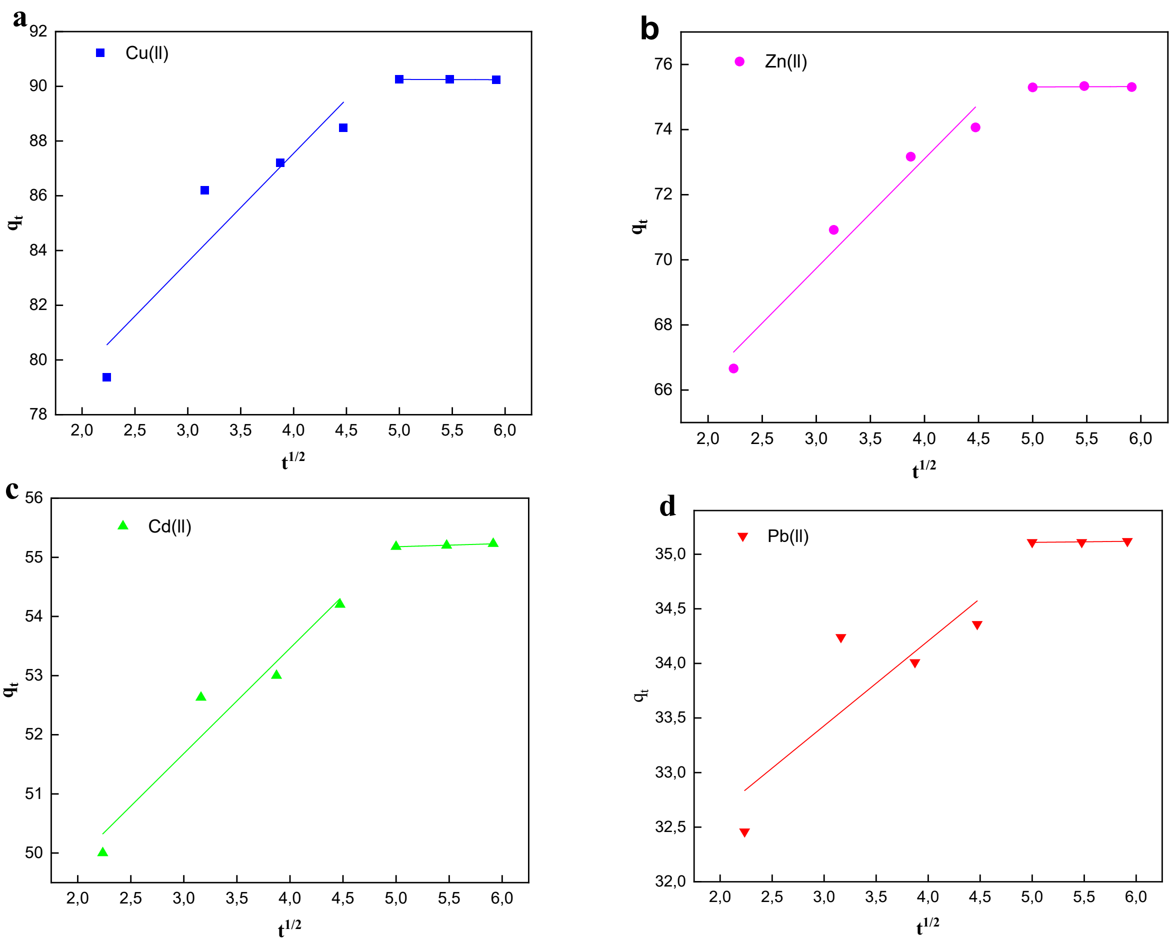

Fig S3a, b, c, d Intra-particle diffusion model (a) copper, (b) zinc, (c) cadmium and (d) lead. Adsorption conditions: V = 10 mL, m = 10 mg of adsorbent, pH = 6, and optimum concentrations: 180.66, 140.10, 99.70 and 95,63.10^{-3} mg L^{-1} for Cu(II), Zn(II), Cd(II) and Pb(II) respectively at 25 °C.

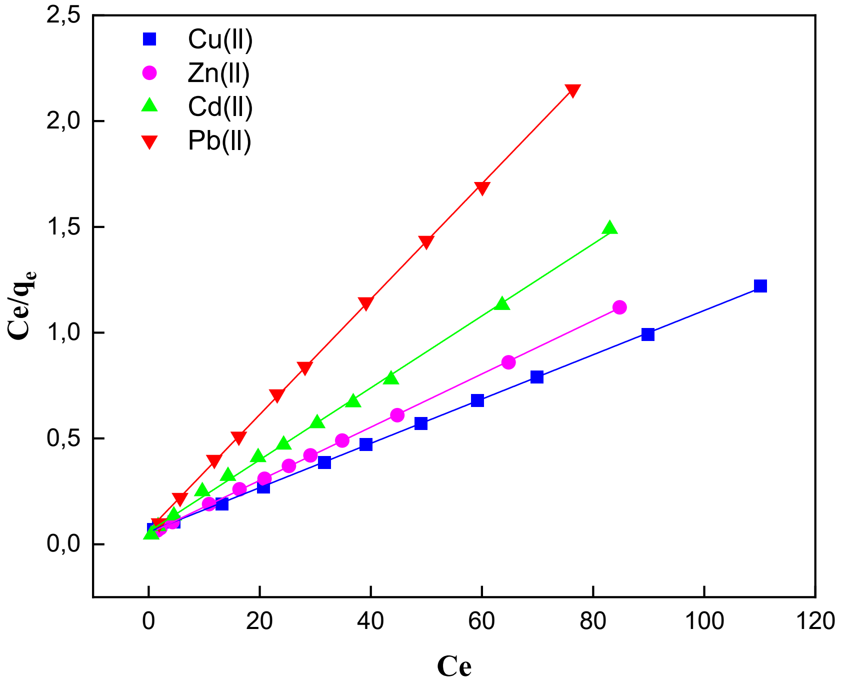

Fig S4 Adsorption isotherms of **SiPy** towards Cu(II), Zn(II), Cd(II) and Pb(II) fitted by Langmuir. Adsorption conditions: m = 10 mg, V = 10 mL, [Mn(II)] = 10 to 300.10^{-3} g L^{-1}, pH = 6, time = 25 min at 25 °C.

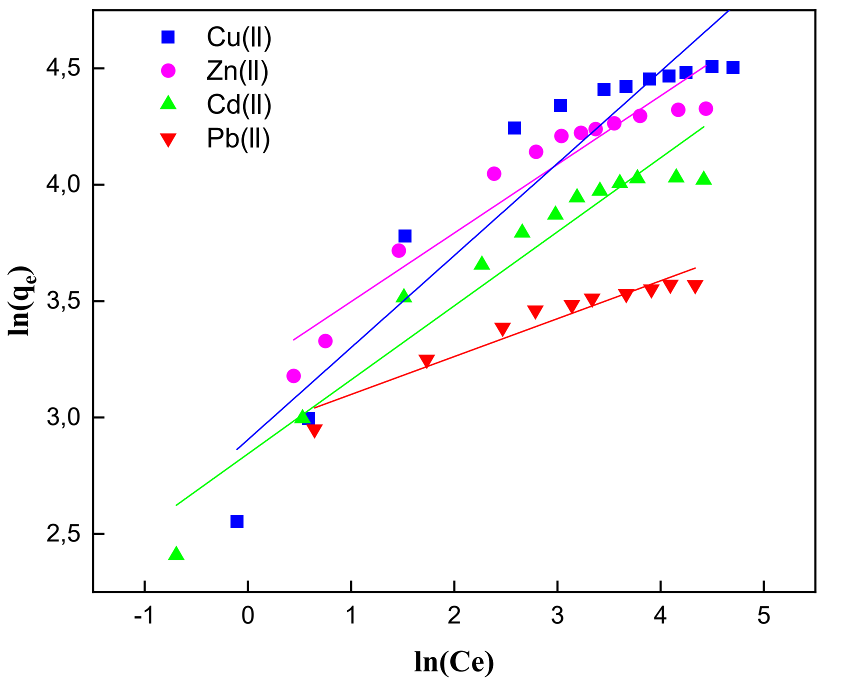

Fig S5 Adsorption isotherms of **SiPy** towards Cu(II), Zn(II), Cd(II) and Pb(II) fitted by Freundlich. Adsorption conditions: m = 10 mg, V = 10 mL, [Mn(II)] = 10 to 300.10^{-3} g L^{-1}, pH = 6, time = 25 min at 25 °C.

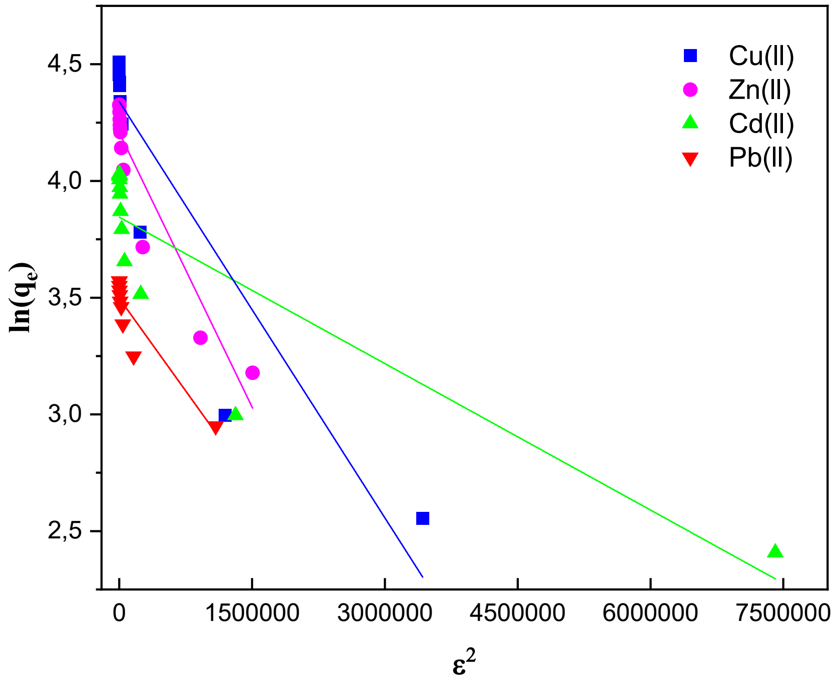

Fig S6 Adsorption isotherms of **SiPy** towards Cu(II), Zn(II), Cd(II) and Pb(II) fitted by Dubinin-Radushkevich (D-R). Adsorption conditions: m = 10 mg, V = 10 mL, [Mn(II)] = 10 to 300.10^{-3} g L^{-1}, pH = 6, time = 25 min at 25 °C.

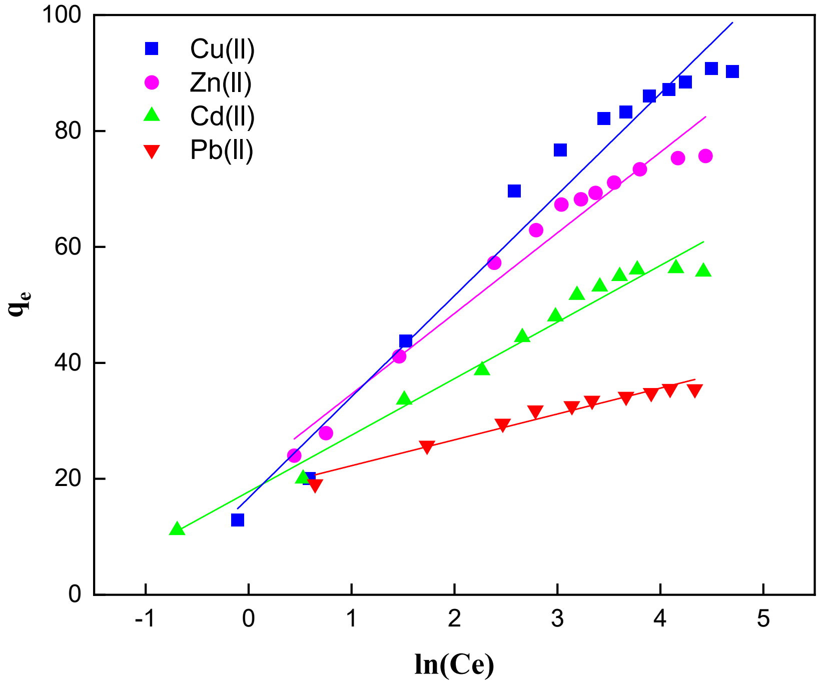

Fig S7 Adsorption isotherms of **SiPy** towards Cu(II), Zn(II), Cd(II) and Pb(II) fitted by Temkin. Adsorption conditions: m = 10 mg, V = 10 mL, [Mn(II)] = 10 to 300.10^{-3} g L^{-1}, pH = 6, time = 25 min at 25 °C.
